# Complete genome sequence of *Neobacillus* sp. strain OS1-2, a denitrifying bacterium isolated from apple orchard soil

**DOI:** 10.1128/mra.01114-24

**Published:** 2024-12-19

**Authors:** Jinwoo Ahn, Jeonghwan Jang

**Affiliations:** 1 Division of Biotechnology, Jeonbuk National University, Iksan, Jeonbuk, South Korea; 2 Advanced Institute of Environment and Bioscience, Jeonbuk National University, Iksan, Jeonbuk, South Korea; California State University San Marcos, San Marcos, California, USA

**Keywords:** soil microbiology, denitrification, nitrogen cycle enzymes

## Abstract

We report here the complete genome sequence of *Neobacillus* sp. strain OS1-2, a bacterium isolated from apple orchard soil and possessing a complete set of denitrification functional genes in its genome. The isolate was observed to perform denitrification under aerobic and anaerobic conditions.

## ANNOUNCEMENT

**Fig 1:**
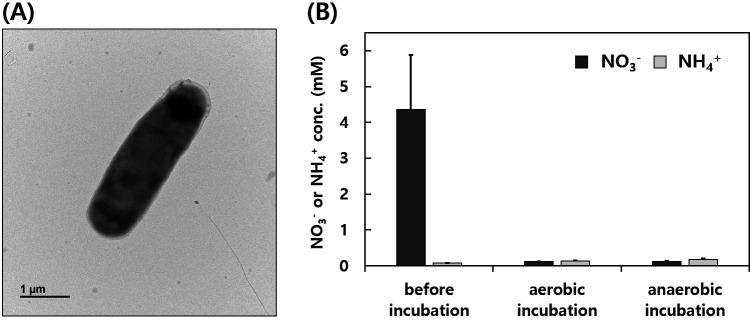
(**A**) A cell image of strain OS1-2 visualized by Hitachi-H7650 transmission electron microscope (Hitachi, Japan), and (**B**) changes in NO_3_^-^ and NH_4_^+^ concentrations in the supernatant of the strain OS1-2 culture after aerobic or anaerobic incubation for 2 days at 30°C.

Denitrifying microbes play critical roles in removing excessive NO_3_^-^ in soil environment and wastewater treatment systems for balanced nitrogen (N) cycle and cleaning water, respectively (1). To study the ecological roles of soil denitrifiers, *Neobacillus* sp. OS1-2 have been isolated from a surface soil sample (0–5 cm depth) collected using a trowel at an apple orchard located in Iksan city, Republic of Korea (35°58′44.03″N 127°3′ 31.33″E) in August 2023. For the isolation of strain OS1-2, 1 g of the soil sample was mixed with 10 mL of phosphate-buffered saline (pH 7.0), and 100 µL of the 1:100 diluted soil suspension was then spread on R2A agar plates (MB cell, Korea) supplemented with 5 mM NO_3_^-^ and 10 mM acetate (R2A-NA), followed by anaerobic incubation for 3 days at 30°C. Typical rod-shaped bacillus morphology of strain OS1-2 was observed under the microscope (Fig. 1A). After incubating strain OS1-2 in 10 mL R2A-NA broth (MB cell, Korea) in normal test tubes (aerobic) or anaerobic culture tubes with N_2_ atmosphere in the headspace (anaerobic) on an orbital shaker (100 rpm) for 2 days at 30°C, NO_3_^-^ was almost completely removed from the culture supernatant without extracellular NH_4_^+^ production regardless of oxygen presence as confirmed by the colorimetric method described previously (Fig. 1B) (2).

Genomic DNA was extracted using the DNeasy PowerLyzer Microbial Kit (Qiagen, USA) for the colonies of strain OS1-2 formed on the agar plate. The genomic DNA sample was submitted to the KNU NGS Center (Kyungbuk National University, Daegu, Republic of Korea) for whole-genome sequencing using the Oxford MinION Nanopore platform (Oxford Nanopore Technologies, UK). Sequencing libraries were created with the Native Barcoding Kit 24 V14, and sequencing was performed on an R10.4.1 flow cell using a MinION Mk1c device, in accordance with the manufacturer’s guidelines. Default parameters were used for all software described in this study unless otherwise specified. A total of 239,169 pass-filter (PF) reads with an *N*_50_ value of 8,124 bp (total 1,648,356,239 bp) were obtained after basecalling and demultiplexing were performed using Guppy Basecaller version 2.24 (3), and reads shorter than 1 kb and the worst 5% of read bases were removed using Filtlong version 0.2.1 (https://github.com/rrwick/Filtlong). The PF reads trimmed for adapter sequences by Trimmomatic version 0.36 (4) were assembled *de novo* into a single contig, circularized, and polished using Flye version 2.9.1 (5). The assembled complete circular genome had a total length of 4,733,117 bp, and genome coverage was estimated to be 348-fold. Gene prediction and annotation were done using the NCBI Prokaryotic Genome Annotation Pipeline version 6.8 (6). Average nucleotide identity (ANI) values were calculated by the Taxonomy Check function of PGAP by comparing the assembled genome to one of the type strains in GenBank (7).

The genome of strain OS1-2 had a G + C content of 40.1% and contained 4,507 protein-coding sequences, 82 pseudogenes, 108 tRNAs, 39 rRNAs (13 5S, 13 16S, and 13 23S), and 5 noncoding RNAs. The most closely related genome of the known type strain was *Neobacillus bataviensis* LMG 21833^T^ (GenBank accession no. GCA_000307875.1) based on the ANI value (88.07% identity with 66.5% query coverage) below the species cutoff value (8), indicating that strain OS1-2 would be a novel species within the genus *Neobacillus*. A complete set of denitrification functional genes was found in the genome of strain OS1-2 (Table 1).

**TABLE 1:** Denitrification functional genes found in the genome of strain OS1-2 (GenBank accession no. NZ_CP133265)

Gene	Enzyme	Locus tag
*narG*	Nitrate reductase	RCG19_RS22825
*nirK*	Nitrite reductase	RCG19_RS06400
*norB*	Nitric oxide reductase	RCG19_RS14055
*nosZ*	Nitrous oxide reductase	RCG19_RS08720

## Data Availability

The complete whole genome sequence of *Neobacillus* sp. OS1-2 has been deposited in GenBank under the accession number NZ_CP133265, and raw read sequence data are available with SRA accession number SRX24710422.
